# Supplementary material for: Cutaneous Delivery and Biodistribution of Cannabidiol in Human Skin after Topical Application of Colloidal Formulations
Source: Pharmaceutics. 2024 Jan 30;16(2):202. doi: 10.3390/pharmaceutics16020202 (PMC10892191; doi:10.3390/pharmaceutics16020202)
Supplement: Supplementary file 1 [file pharmaceutics-16-00202-s001.zip › pharmaceutics-2802780-supplementary.pdf]

pharmaceuticals

Supplementary information to manuscript

Cutaneous delivery and biodistribution of cannabidiol in human skin after topical application of colloidal formulations

Maria Lapteva [1,2], Jonathan Faro Barros [1,2], and Yogeshvar N. Kalia [1,2]*

[1] School of Pharmaceutical Sciences, Institute of Pharmaceutical Sciences of Western Switzerland, University of Geneva, CMU-1 rue Michel Servet, 1211 Genève 4, Switzerland.
[2] Institute of Pharmaceutical Sciences of Western Switzerland, University of Geneva, CMU-1 rue Michel Servet, 1211 Genève 4, Switzerland.

* Correspondence: Yogi.Kalia@unige.ch; Tel.: +41 (0)22 379 3355

1. Preparation of standards, samples and other solutions

1.1. Mobile phases

Mobile phases were prepared with HPLC grade solvents and MilliQ grade water. Solvent A consisted of MilliQ water and Solvent B was a mixture of ACN:MeOH (50:50) containing 0.1% FA (according to Knaub et al. 2019 [1]).

1.2. Calibration and quality control solutions

All solution and samples were prepared "mass to mass" to avoid any imprecision due to organic solvent pipetting. All masses were noted and accounted for in the final calculations.

Calibration standards of 1, 2, 4, 10, 20, 40, 60, 80 and 100 ng/g were prepared by dilution of stock solutions prepared either in MeOH:water 90:10 (sample diluent A) or PBS + Tween 80 0.5% (sample diluent B). Quality control solutions of 2, 4, 60 and 100 ng/g were prepared and injected on three different days. These solutions served for method validation.

2. Preliminary study – CBD solubility screening

In order to ensure appropriate sink conditions, the receiver compartment of the Franz cell must ensure sufficient CBD solubilisation. The solubility of CBD was determined in the following receiver receptor media: (i) PBS + 0.5 % Tween 20, (ii) PBS + 1% Tween 20, (iii) PBS + 0.5% Tween 80, (iv) PBS + 1% Tween 80, (v) PBS + 0.5% Brij C20, and (vi) PBS + 1% Brij C20. Approximately 5 mg of CBD were dispersed in 1 mL of each medium and left under stirring at 32°C for 24 h in triplicate. The excess of powder was removed by centrifugation (15 min, 10 000 rpm), while the supernatant underwent CBD quantification. The solubility results are shown in **Figure S.1**:

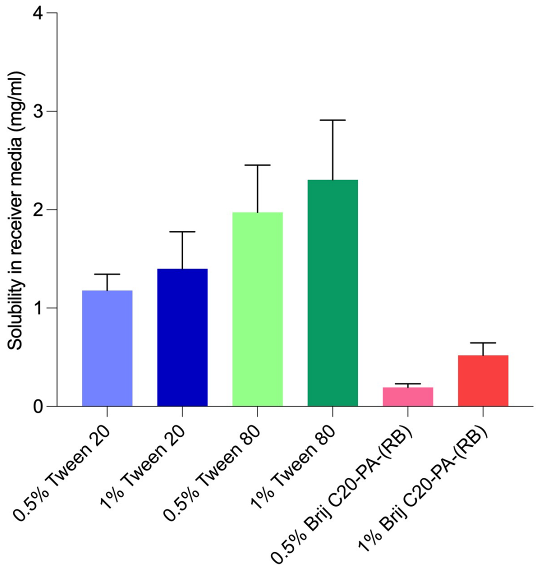

Figure S.1: Solubility of CBD in different receiver media (mean ±SD; n=3).

All Tween-based receiver phases were able to solubilise CBD at the minimum required concentration, 0.5 mg/g. Tween 80 showed the highest ability to solubilise CBD: the solubility was 1.97 ± 0.48 mg/g in the 0.5% solution and 2.30 ± 0.61 mg/g in the 1% solution. To minimise the surfactant content, it was decided to work with PBS + 0.5%Tween 80.

3. Extraction of CBD from skin samples

The extraction method was validated by injecting a known amount of CBD intradermally in a Ø1 cm full-thickness skin sample. Injected doses ranged between 2.5 and 15.7 µg of CBD. The skin samples were then frozen at -20 °C prior to being cut in 1 x1 mm pieces and immersed in 10 mL of MeOH:water 90:10. CBD was extracted for 4 h at RT under stirring. After centrifugation, appropriate dilution and addition of CBD-d3, CBD was quantified by LC-MS/MS in the extracts.

The extraction method was considered as valid since 98.8 ± 22.9% of CBD was recovered upon skin extraction (mean ± SD, n=8)

4. Stability of CBD in the receiver compartment

CBD dissolved in PBS + 0.5% Tween 80, at 60 ng/g was placed in contact with the dermal side of human skin samples and placed at 32 °C for 48 h. The solution was sampled at regular time intervals. After centrifugation, appropriate dilution and addition of CBD-d3, CBD was quantified by LC-MS/MS. No known degradation compounds were detected. The relative amount of CBD recovered as a function of time is presented in **Figure S.2**.

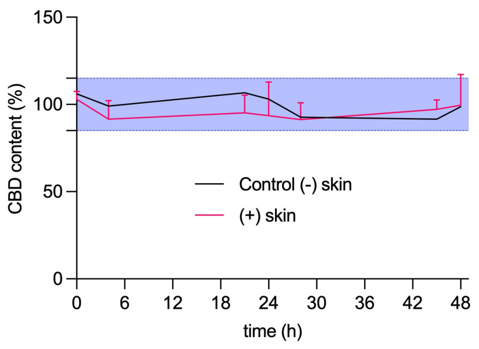

Figure S.2: CBD content over time when in contact with the dermal side of human skin at 32°C (mean ±SD, n = 6 for the test group and n = 1 for the control group). The blue area denotes the acceptance range (± 15%).

At the end of the experiment, 99.5 ± 17.7% of CBD was recovered. According to the obtained data, it was concluded that CBD is stable in contact with skin at 32°C for at least 48 h, thus allowing us to proceed with the skin permeation experiments.

5. Analytical method validation

The validation of the analytical method was performed as per current EMA [2] and ICH guidelines [3]: the specificity, sensitivity, linearity, accuracy and precision were tested with the aim of quantifying CBD in skin extracts and permeation samples. All experiments were performed with human skin samples obtained shortly after surgery from the Department of Plastic, Aesthetic and Reconstructive Surgery, Geneva

University Hospital (Geneva, Switzerland), fatty tissue was removed and the skin was stored at −20 °C. The donation was approved by the Cantonal Committee for Ethics in Research (CCER: 2021-01578).

5.1. Specificity

ICH defines specificity as “the ability to assess unequivocally the analyte in the presence of components which may be expected to be present. Typically, this might include impurities, degradants, matrix, etc.”

5.1.1. Selection of transitions and potential degradation products

Four different ion transitions were followed in MRM (Multiple reaction monitoring) mode: two transitions for CBD and two for its deuterated internal standard CBDd3.

Figure S.3 shows MRM (Multiple reaction monitoring) traces obtained after the injection of a CBD standard of 100 ng/g and CBD-d3 standard of 100 ng/g. As expected CBD and CBD-d3 co-eluted at a retention time of 2.27 min. However, there was no signal from transitions 318.30 > 123.06 and 318.30 > 196.20 in the CBD standard and there was no signal from transitions 315.23 > 123.00 and 315.23 > 193.14 in the CBD-d3 standard, meaning that the MS/MS detection was selective for both compounds.

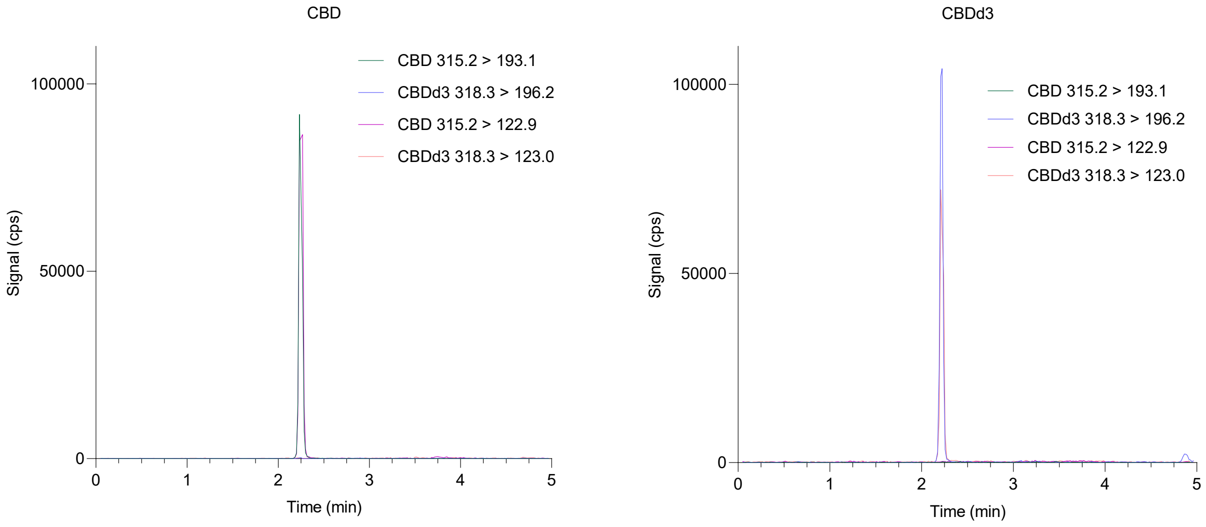

Figure S3: MRM traces of a CBD standard of 100 ng/g (left panel) and CBD-d3 standard of 100 ng/g (right panel).

Figure S.4 shows MRM (Multiple reaction monitoring) for a blank sample, Tween 80 solution 1%, blank permeation sample and blank extraction sample. Consistent with the chromatograms obtained in UV detection, there were no interfering peaks in those negative control samples.

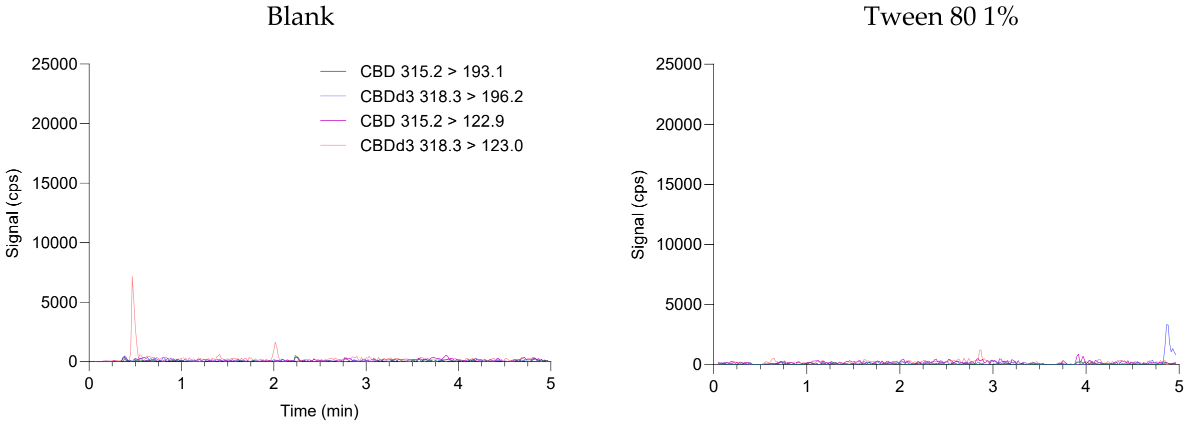

Copyright: © 2024 by the authors. Licensee MDPI, Basel, Switzerland. This article is an open access article distributed under the terms and conditions of the Creative Commons Attribution (CC BY) license (https://creativecommons.org/licenses/by/4.0/).

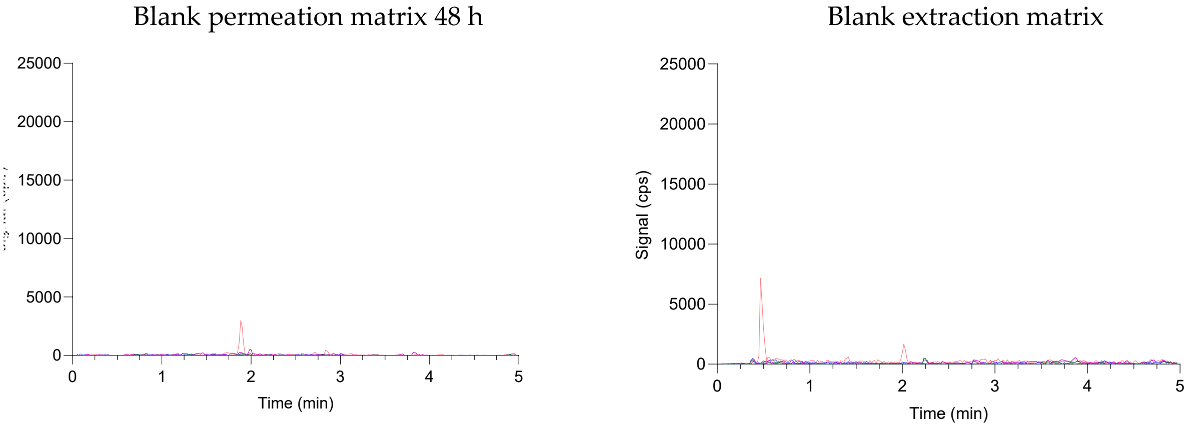

Figure S.4: MRM traces of a blank sample, Tween 80 solution 1%, blank permeation sample and blank extraction sample.

It has been reported that CBD is prone to convert to THC and/or CBG under acidic conditions [4]. Standards of THC and CBG were injected. **Figure S.5** shows MRM traces of THC and CBG standards.

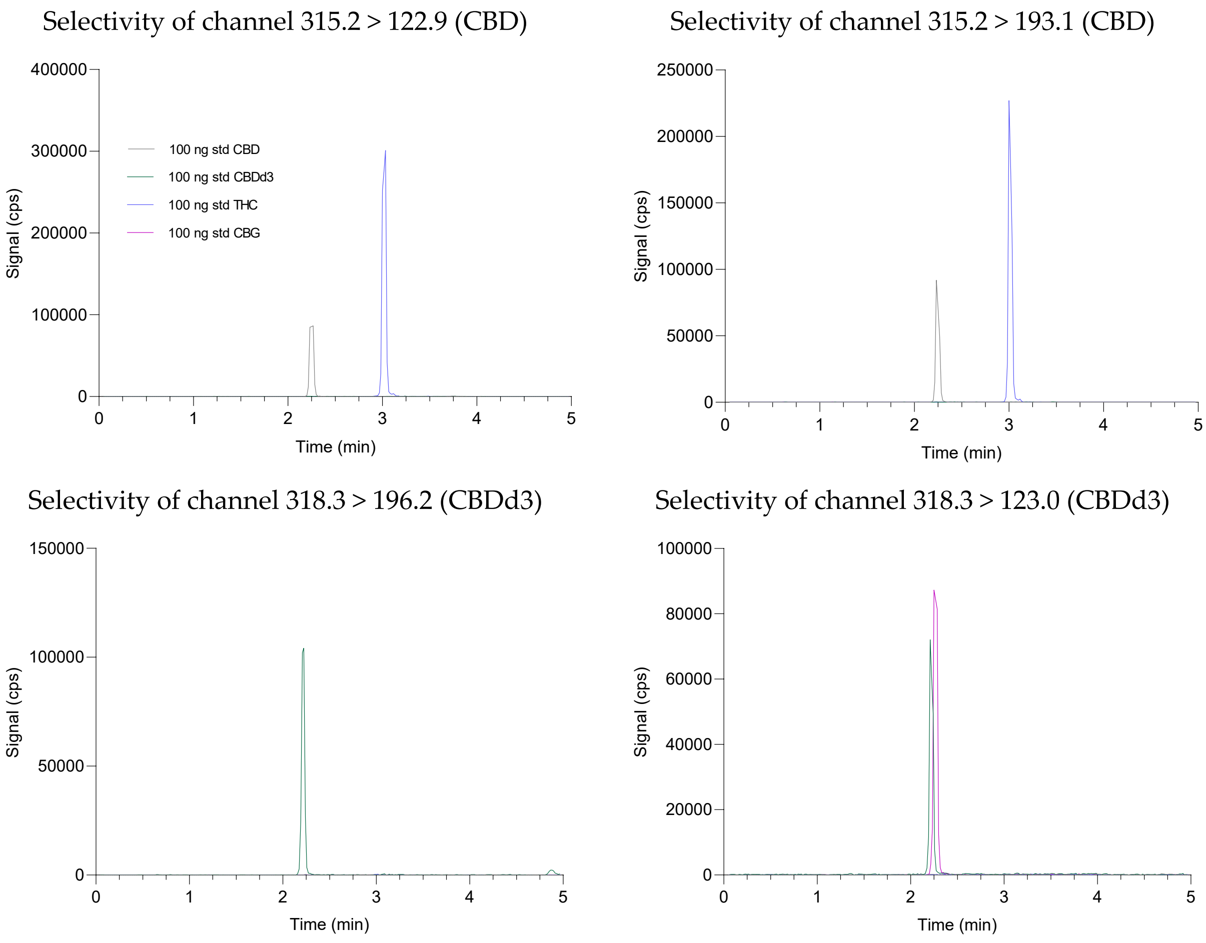

Figure S.5: Selectivity of the different MRM channels for CBD, CBDd3, THC and CBG.

THC is a degradation product of CBD originating from an intramolecular cyclization, therefore it has the same MW as CBD and thus the same precursor ion. Given the similarity of both compounds the fragmentation pattern in the collision cells also generates the same product ion fragments. This explains the fact that THC can be monitored using both 315.23 > 123.00 and 315.23 > 193.14 transitions. However, it elutes at 3.04 min, which means that the method retains is specificity thanks to the separative LC method.

On the other hand, CBG resulted in a peak that could be monitored using the CBDd3 transition 318.30 > 123.06 and eluted closely to the CBDd3 peak itself. In order to ensure the specific detection of the internal standard, the transition 318.30 > 123.06 was abandoned as it could not maintain specificity.

In conclusion, transition 315.23 > 193.14 was used to quantify CBD and transition 318.30 > 196.20 was used to quantify CBDd3.

5.1.2. Matrix effect on Electrospray Ionisation (ESI).

The matrix effect often occurs during electrospray ionisation as co-elution of the sample matrix with the analyte impairs its ionization. Since the internal standard was deuterated CBD, it possessed the same physicochemical properties as CBD. Thus, it co-eluted with CBD and underwent the same matrix effect, enabling any possible matrix effect during quantification to be taken into account.

5.2. Linearity

Calibration curves were constructed by plotting the ratio of CBD and CBD-d3 peak area (cps.min) against the ratio of respective nominal concentrations (ng/g). A good linear fit was found in the concentration range of 1 – 100 ng/g. Correlation coefficients for all calibration curves were found to be superior to 0.98. **Figure S.6** shows an example of the obtained calibration curves.

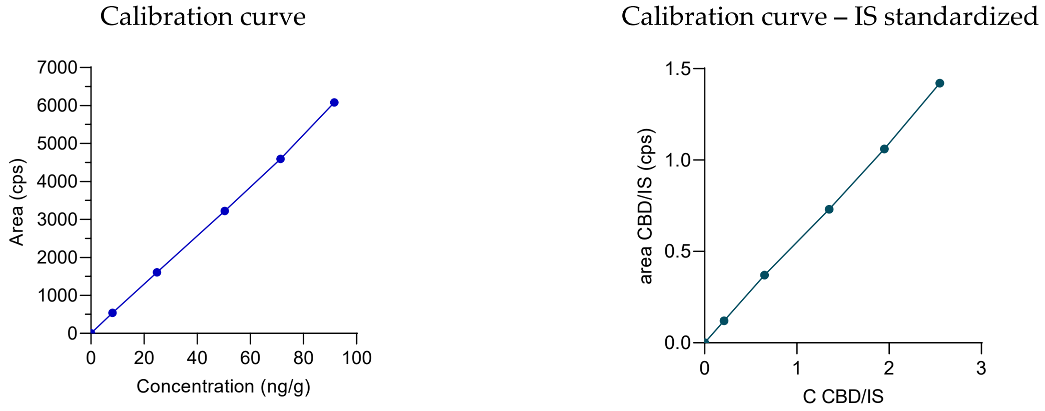

Figure S.6: Calibration curves – Peak area as a function of concentration (left panel); Internal standard standardized peak area as a function of relative concentrations (right panel).

For the quantification of extraction and permeation samples, calibration curves were prepared in MeOH:water 90:10 (sample diluent A; extraction solvent) and PBS + Tween 80 0.5% (sample diluent B; receiver medium) respectively.

5.3. Precision and Accuracy

The accuracy of an analytical method describes the closeness of the experimentally determined concentration obtained by the method to the nominal concentration of the analyte (expressed in percentage). The precision of the analytical method describes the closeness of repeated individual measures of analyte. Precision is expressed as the coefficient of variation (CV or RSD).

Precision and accuracy were assessed using quality control solutions containing 1, 2, 4, 20, 60 and 100 ng/g of CBD and approximately 40 ng/g of CBD-d3 as internal standard in both extraction and permeation matrixes. For the method to be considered as accurate, the mean concentration should be within 15% of the nominal values for the QC samples, except for the LLOQ which should be within 20% of the nominal value. For the method to be considered as precise, RSD value should not exceed 15% for the QC samples, except for the LLOQ which should not exceed 20%.

Table S.1 summarizes the data obtained. Data highlighted in green denotes acceptable recovery (85-115%) and hence Accuracy of the method. The RSD (precision) should be less than 15% (also in green).

Table S.1: Accuracy and precision

		Validation in EXTRACTION MATRIX					
		Intra-day (n=3)			Inter-day (n=9)		
Target [CBD] (ng/g)	Nominal [CBD] (ng/g)	measured [CBD] (mean ± SD in ng/g)	Accuracy in % (measured [CBD] / Nominal [CBD])	Precision (RSD in %)	measured [CBD] (mean ± SD in ng/g)	Accuracy in % (measured [CBD] / Nominal [CBD])	Precision (RSD in %)
2	2.31	2.1 ± 0.1	91.1%	4.3%	2.3 ± 0.2	101.7%	9.7%
20	22.06	20.7 ± 0.9	93.9%	4.5%	22.4 ± 2.5	97.9%	11.3%
100	101.61	109.2 ± 2.1	107.5%	2.0%	105.4 ± 4.1	104.3%	3.8%

		Validation in PERMEATION MATRIX									
		Intra-day (n=3)					Inter-day (n=9)				
Target [CBD] (ng/g)	Nominal [CBD] (ng/g)	measured [CBD] (mean ± SD in ng/g)			Accuracy in % (measured [CBD] / Nominal [CBD])	Precision (RSD in %)	measured [CBD] (mean ± SD in ng/g)			Accuracy in % (measured [CBD] / Nominal [CBD])	Precision (RSD in %)
1	1.15	1.14	±	0.16	99.5%	13.9%	1.14	±	0.20	99.6%	17.7%
4	3.64	3.6	±	0.1	99.0%	2.4%	3.6	±	0.3	99.3%	9.3%
20	18.68	19.2	±	1.0	103.0%	5.2%	18.7	±	1.0	98.5%	5.4%
100	97.63	104.9	±	2.3	107.4%	2.2%	91.0	±	11.4	94.6%	12.5%

In both matrices and for all QC solutions, both in intra-day (or "within run") and in inter-day (or "between run") trials resulted in a recovery (accuracy) ranging between 85 and 115 %; the RSDs were all below 15% for QC solutions higher than the LLOQs. Only the Inter-day precision at the LLOQ in the permeation matrix (17.7%) was above 15% but below 20%, thus also in the acceptable range. **Therefore, the method could be considered as Accurate and Precise.**

5.4. Limit of detection and limit of quantification

The lowest amounts to be detected (LLOD) and lowest limit of CBD quantified (LLOQ) were different for both matrices and are presented in **Table S.2**. The LLOQs were validated with respect to precision and accuracy

Table S.2. LOD and LOQ values

	In extraction matrix	In permeation matrix
LLOD (ng/g)	0.7	0.4
**LLOQ (ng/g)**	**2.3**	**1.2**

Given the previously mentioned data, the method was considered as valid in the range of 2.3 to 100 ng/g for skin extraction samples and in the range of 1.2 to 100 ng/g for skin permeation samples.

6. References

1. Knaub, K., et al., *A Novel Self-Emulsifying Drug Delivery System (SEDDS) Based on VESIsorb(®) Formulation Technology Improving the Oral Bioavailability of Cannabidiol in Healthy Subjects.* Molecules, 2019. **24**(16).
2. EMA, *Guideline on bioanalytical method validation*. 2011.
3. ICH, *ICH M10 on bioanalytical method validation*. 2019.
4. Layton, C., J. Runco, and A. Aubin. *WATERS application note: Forced degradation of Cannabidiol*. 2016 11.07.22]; Available from: https://www.google.com/url?sa=t&rct=j&q=&esrc=s&source=web&cd=&cad=rja&uact=8&ved=2ahUKEwi0rtrM5vD4AhUOhYkEHXgRAsIQFnoECAMQAQ&url=https%3A%2F%2Fwww.waters.com%2Fwebassets%2Fcms%2Flibrary%2Fdocs%2F720005766en.pdf&usg=AOvVaw19pmcHPitJSQyXq4FJe6Rm.

Disclaimer/Publisher's Note: The statements, opinions and data contained in all publications are solely those of the individual author(s) and contributor(s) and not of MDPI and/or the editor(s). MDPI and/or the editor(s) disclaim responsibility for any injury to people or property resulting from any ideas, methods, instructions or products referred to in the content.
